# Functional differences between protamine preparations for the transfection of mRNA

**DOI:** 10.1080/10717544.2020.1790692

**Published:** 2020-08-17

**Authors:** Natalia Teresa Jarzebska, Severin Lauchli, Christoph Iselin, Lars E French, Pal Johansen, Emmanuella Guenova, Thomas M Kündig, Steve Pascolo

**Affiliations:** a Department of Dermatology, University Hospital of Zürich, Zürich, Switzerland; b Faculty of Science, University of Zürich, Zürich, Switzerland; c Faculty of Medicine, University of Zürich, Zürich, Switzerland; d Department of Dermatology and Allergy, University Hospital, LMU Munich, Munich, Germany; e Lausanne University Hospital (CHUV), Lausanne, Switzerland; f Faculty of Biology and Medicine, University of Lausanne, Lausanne, Switzerland

**Keywords:** Protamine, nanoparticles, RNA, proticle, transfection, toll-like receptor

## Abstract

Protamine is a natural cationic peptide mixture used as a drug for the neutralization of heparin and in formulations of slow-release insulin. In addition, Protamine can be used for the stabilization and delivery of nucleic acids (antisense, small interfering RNA (siRNA), immunostimulatory nucleic acids, plasmid DNA, or messenger RNA) and is therefore included in several compositions that are in clinical development. Notably, when mixed with RNA, protamine spontaneously generates particles in the size range of 20–1000 nm depending on the formulation conditions (concentration of the reagents, ratio, and presence of salts). These particles are being used for vaccination and immuno-stimulation. Several grades of protamine are available, and we compared them in the context of complex formation with messenger RNA (mRNA). We found that the different available protamine preparations largely vary in their composition and capacity to transfect mRNA. Our data point to the source of protamine as an important parameter for the production of therapeutic protamine-based complexes.

## 1. Introduction

Protamine is a conserved natural cationic peptide mixture that condenses sperm DNA in all vertebrates (Balhorn, 2007). Unrelated to its physiological role, protamine is used as a drug to inhibit heparin after surgery (Jaques, 1973). It is also the moiety that mediates slow release in intermediate-acting insulin formulations. In addition, protamine has been reported to enhance the transfection of RNA in cultured cells (Amos, 1961; Amos and Kearns, 1963; Billiau et al., 1969) and is still intensively used to deliver different types of RNA *in vitro* and *in vivo* (Amos, 1961; Amos and Kearns, 1963; Choi et al., 2010; Bell et al., 2018; Sato et al., 2018; Shi et al., 2019). Indeed, protamine spontaneously associates with purified, recombinant, or chemically synthesized nucleic acids and forms complexes (Amos and Kearns, 1963; Billiau et al., 1969; Hoerr et al., 2000; Scheel et al., 2004; Scheel et al., 2005; Scheel et al., 2006). We showed that nanoparticles of a defined size ranging from 20 nm to more than 1000 nm can be easily obtained by simply adjusting the concentration, formulation, and ratio of RNA and protamine (Rettig et al., 2010; Tusup and Pascolo, 2017). Such protamine-based nanoparticles (also termed proticles) are broadly used to deliver oligonucleotides (antisense, small interfering RNA (siRNA), and immunostimulatory nucleic acids; reviewed by Scheicher et al. (2015)) as well as encoding nucleic acids (plasmid DNA (Vighi et al., 2012) or messenger RNA (mRNA) (Hoerr et al., 2000; Scheel et al., 2004; Scheel et al., 2005; Scheel et al., 2006; Weide et al., 2009; Parvanova et al., 2011); reviewed by Kauffman et al. (2016)). Furthermore, complexing protamine with antibodies offers a platform to associate RNA and antibodies (Baumer et al., 2016; Baumer et al., 2015; Song et al., 2005). Because of the crucial role this component plays in drug development, we aimed to compare different sources of protamine. Indeed, it is available in hydrochloric or sulfated forms and originates from herring or salmon. In addition, as a biochemical product, protamine is available in different purity grades. The impact of the origin of protamine on particle formation and functionality has not been explored. We report here that although all protamine sources can form similar homogenous nanoparticles when mixed with RNA, and produce immunostimulatory complexes, the pharmaceutical grade chloride protamine used for heparin neutralization is the best for obtaining the expression of condensed mRNA.

## 2. Materials and methods

### 2.1. mRNA

mRNAs were produced using *in vitro* transcription at the ‘ivt mRNA production and formulation platform’ in Zürich (http://www.cancer.uzh.ch/en/Research/mRNA-Platform.html). The 5′ end consisted of a CleanCap^TM^ (Trilink) followed by an eIF4G aptamer as the 5′ untranslated region (Tusup and Pascolo, 2018) and by a codon-optimized firefly luciferase open reading frame. The 3′ end consisted of a tandem repeat of the mouse beta-globin 3′ UTR and a poly-A tail (Tusup et al., 2019). The transcription was made in the presence of the four canonical bases (alanine(A), cysteine (C), glycine (G) , and uracil (U)) to obtain immunostimulatory RNA and in the presence of pseudo-uridine instead of uridine to obtain immuno-silent mRNA used for luciferase expression assays. RNA was diluted in RNase-free water, and the concentration measured using a nanodrop was adjusted to 1 mg/ml. The quality and integrity of ivt mRNAs were checked using agarose gel electrophoresis. The RNAs were stored at −20 °C.

### 2.2. Protamines

We compared the performance of eight commercially available protamines: clinical protamine Ipex 5000 IU (MEDA) that is a solution, and seven Sigma-Aldrich products in powders: protamine grade II–V, protamine grade X, protamine meeting USP standards (Sigma-Aldrich, St. Louis, MO) and protamine for biochemistry. The protamine sources and formulations are shown in Table 1.

**Table 1.** Formulations and sources of protamines used in this article.

Protamine type	Source	Salt
Clinical 5000	Salmon	Chloride
Grade II	Salmon	Sulfate
Grade III	Herring	Sulfate
Grade IV	Salmon	Not indicated
Grade V	Salmon	Chloride
Grade X	Salmon	Sulfate
Grade USP	Salmon	Sulfate
Grade for Biochemistry	Not indicated	Sulfate

All protamines were diluted in pure water to a concentration of 1 mg/ml and stored at 4 °C.

### 2.3. Sodium dodecyl sulfate-polyacrylamide gel electrophoresis (SDS-PAGE)

SDS-PAGE was carried out using 5–20% polyacrylamide gels. Five micrograms of each Protamine type solution was mixed with 2x NuPAGE LDS sample buffer (Invitrogen, Carlsbad, MA) and 1 M dithiothreitol (DTT) solution at a ratio of 5:4:1 (sample in water:LDS:DTT). Samples were loaded on the gel and subjected to electrophoresis. After being separated, the proteins were visualized by staining with GelCode™ blue safe protein stain (Thermo Fisher Scientific, Waltham, MA).

### 2.4. Preparation and physical analysis of protamine-RNA particles

Preparation of the proticles was performed as described previously (Tusup and Pascolo, 2017). In brief, protamine (0.75 mg/ml) was mixed with mRNA (0.25 mg/mL) in equal volumes to obtain a ratio of 3:1 (protamine:mRNA *w/w*) and left for 10 min at room temperature. One milliliter of 5% glucose solution in pure water was added, and the size distribution of the protamine-mRNA complexes was characterized by dynamic light scattering with a ZetaSizer 3000HSA (Malvern Instruments, Worcestershire, UK). Data were analyzed with the built-in DTS software (Raleigh, NC).

### 2.5. Cells and transfections

Human embryonic kidney (HEK) cells were maintained in Roswell Park Memorial Institute (RPMI) medium (Thermo Fisher Scientific) containing 10% fetal calf serum (FCS), 200 mM L-glutamine (Gibco, Carlsbad, CA) and 0.2% antimicrobial reagent Normocin (InvivoGen, San Diego, CA). For Luciferase experiments, the transfection of tumor cells was performed with 100,000 tumor cells per well in 100μl of RPMI medium supplemented with 10% FCS, 200 mM L-glutamine (Gibco) and 0.2% antimicrobial reagent Normocin (InvivoGen) by adding the mixture of RNA and protamine to obtain 1 µg of mRNA per well. The luciferase activity was recorded one day after transfection by adding 25 μl of Bright-Glo luciferase assay solution (Promega, Madison, WA) and measuring the activity using GloMax luminometer equipment (Promega).

### 2.6. Immuno-stimulation

Human PBMCs were isolated from the blood of healthy donors by using Ficoll-Paque™ Plus (GE Healthcare Life Sciences, Chicago, IL). A total of 100,000 cells per well were plated on 96-well plates and incubated with protamine-mRNA nanoparticles overnight. After that time, supernatants were taken, and interferon alpha (IFN-α) concentration was measured via enzyme-linked immunosorbent assay (ELISA) by following the manufacturer’s protocol (Human IFN-α pan ELISA development kit, MABTECH, Sweden). The absorbance was measured on an ELISA reader (BioTek, ELx808 Absorbance Reader, software Gen 5, v2.07 version, Winooski, VT).

### 2.7. Fractionation of clinical protamine

Clinical protamine (2.5 ml at 50 mg/ml) was fractionated with a PD-10 desalting column (GE Healthcare) using a gravity protocol and pure water for elution. Twenty-four fractions were collected and visualized with SDS-PAGE. Subsequently, the protein concentration of all samples was measured with a BCA assay (Thermo Scientific™ Pierce™ BCA™ Protein Assay). The protein concentration in all fractions was adjusted to 90 µg/ml, and the fractions were mixed with mRNA at a ratio of 3:1 (protamine:RNA, *w/w*). The transfection efficiency was measured in HEK cells by luciferase assay, as described above.

## 3. Results and discussion

**Figure 1.**
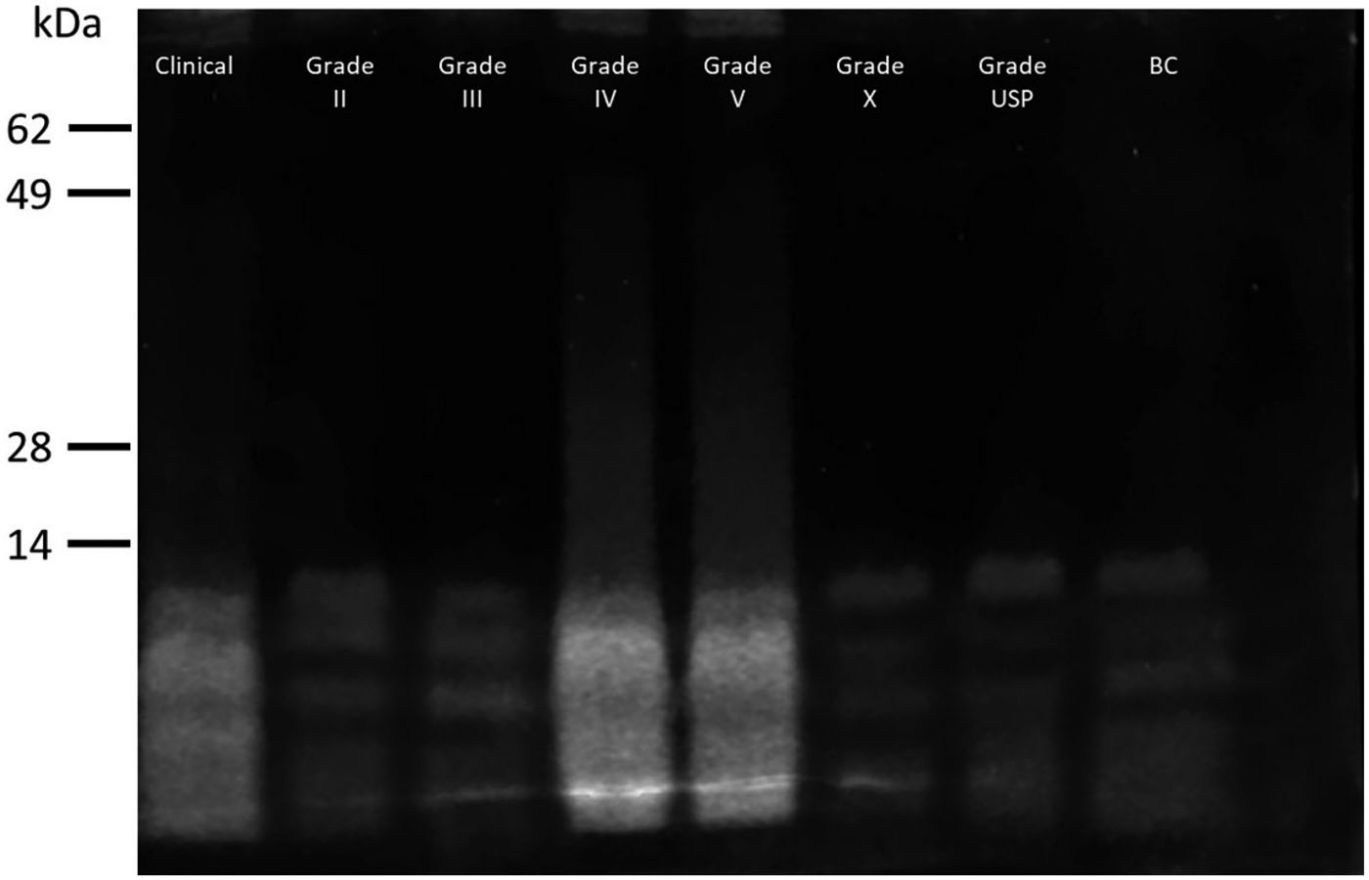
SDS-PAGE analysis. Each protamine formulation was mixed with loading buffer and 5 μg of the sample was run on the gel, which was then stained. The labels indicate the location of bands from a protein marker.

The commercially available protamines (clinical and research grades) were checked for their peptide composition using electrophoresis on SDS-PAGE gels, as shown in Figure 1. Two distinct profiles were obtained: (i) a smear of strongly stained small peptides for clinical hydrochloride protamine and Sigma grades IV and V, and (ii) a few defined weakly stained single bands for Sigma grades II, III X, USP, and BC (in each lane there are 5 μg of protein). The differences in staining patterns and intensities correlated with the counter ion: chloride protamines (Clinical and Grade V) gave the strongly stained smear while sulfate protamines (Grade II, III, X, USP, and BC) gave the weakly stained bands. A direct comparison between clinical grade sulfate and chloride protamines showed that indeed the chloride protamine gives a stronger staining than the sulfate protamine (Supplementary Figure 1). In line with this observation, Benayahu and Aronson (1983) reported different behaviors of chloride and sulfate protamines that could be actually attributed more to the different manufacturing procedure involved in their preparation than to the counterion itself. There was no correlation between the protamine source and its appearance on the SDS-PAGE gel. Thus, several physically distinct products are sold as protamines and these differences prompted us to check their functionality.

First, their capacity to generate nanoparticles when mixed with mRNA in conditions suitable to generate less than 200 nm complexes was evaluated. At this stage, by comparing the concentrations of clinical protamine to those of Sigma protamines, we found that the concentration of clinical protamine is three-fold greater than previously reported (Tusup and Pascolo, 2017). This is due to the fact that the BSA used as a standard in BCA assays has an amino acid composition that is not similar to that of arginine-rich protamine. When BSA standard curves are used, the clinical protamine 5000 has an apparent concentration of 14 mg/ml. We used to dilute it 14 times to obtain a 1 mg/ml solution (Tusup and Pascolo, 2017). However, when the Sigma protamine powder is dissolved at 1 mg/ml and used as a standard in the BCA assay, the clinical protamine 5000 appears to have a concentration of 50 mg/ml (Supplementary Figure 2). Thus, the protamine-to-RNA mass ratio that we used in the past to generate nanoparticles was not 1:1 but 3:1.

**Figure 2.**
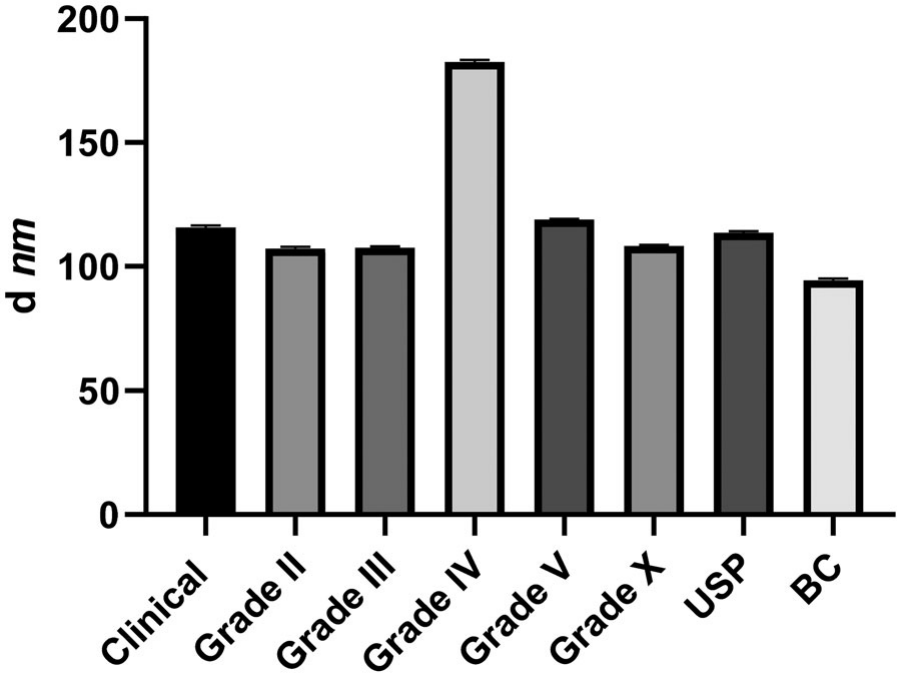
Average size of the protamine-RNA nanoparticles. Protamine and RNA were mixed as specified in the section ‘Materials and Methods’ and the particles were analyzed on a ZetaSizer. The data represent triplicate mean values; error bars: SD; *d* is ‘diameter.’

In light of the new quantification results, we now use a 50-fold dilution of clinical chloride Protamine 5000 to get a concentration of 1 mg/ml and from this solution, we use a Protamine-to-RNA mass ratio of 3:1. Under these conditions, all tested protamines except grade IV could generate nanoparticles of the expected average size close to 100 nm (Figure 2). As the salt of grade IV is not indicated in its documentation, we postulate that this preparation contains a high salt concentration, which results in increased particle sizes when it is mixed with RNA (Rettig et al., 2010).

**Figure 3.**
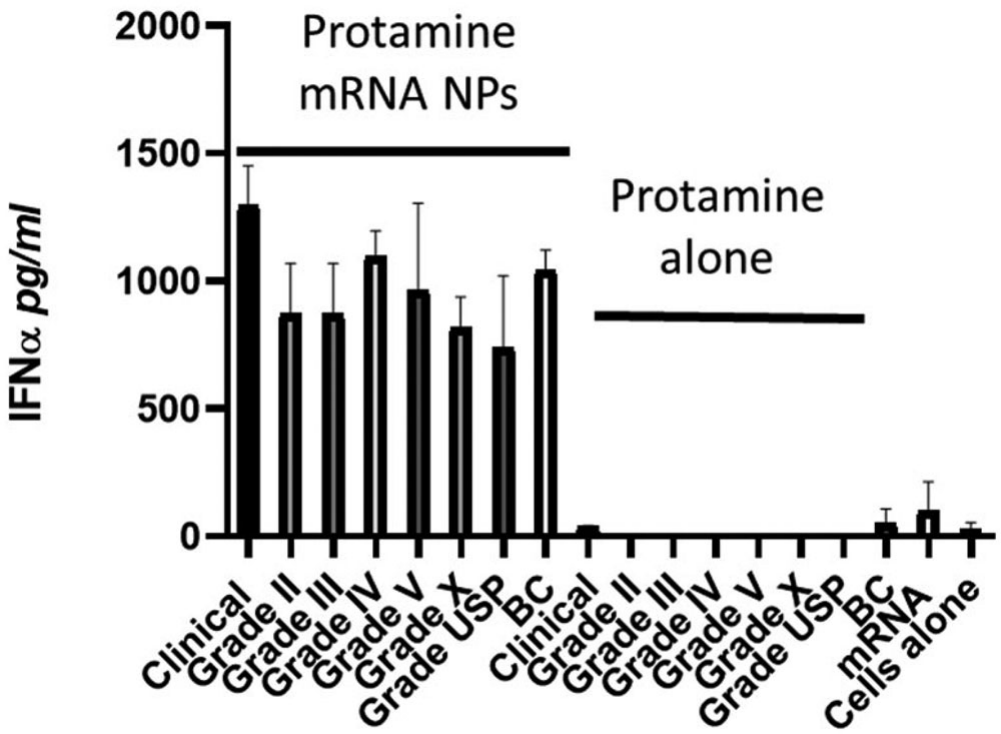
Immunostimulatory features of various types of proticles. Human PBMCs were cultivated for 24 h in the presence of either proticles (‘protamine-mRNA NPs,’ 1 µg of mRNA, and 3 µg of protamine per well) or protamine alone (3 µg per well). Supernatants were analyzed by ELISA for their IFN-α content. Data represent triplicate mean values; error bars: SD.

We have shown that protamine-RNA nanoparticles are immunostimulatory, as they can trigger RNA sensors in endosomes (e.g. toll-like receptors (TLR)- 7 and 8) (Scheel et al., 2006; Scheel et al., 2004; Scheel et al., 2005; Rettig et al., 2010). We also reported previously that the size of the particles affects the type of immuno-stimulation: small particles activate TLR7-expressing IFN-α-producing plasmacytoid dendritic cells, whereas large particles activate TLR8-expressing monocytes that produce inflammatory cytokines such as TNF-α (Rettig et al., 2010). Here, as we focused on particles smaller than 200 nm, we measured IFN-α production in cultured human PBMCs. When mixed with RNA, all commercially available protamines tested here generated similar innate immune responses in PBMCs (Figure 3). Thus, independent of their origin, salt and appearance on SDS-PAGE, protamines when mixed with RNA can generate nanoparticles that can spontaneously home to endosomes and stimulate TLRs, allowing the production of type I interferon by immune cells.

**Figure 4.**
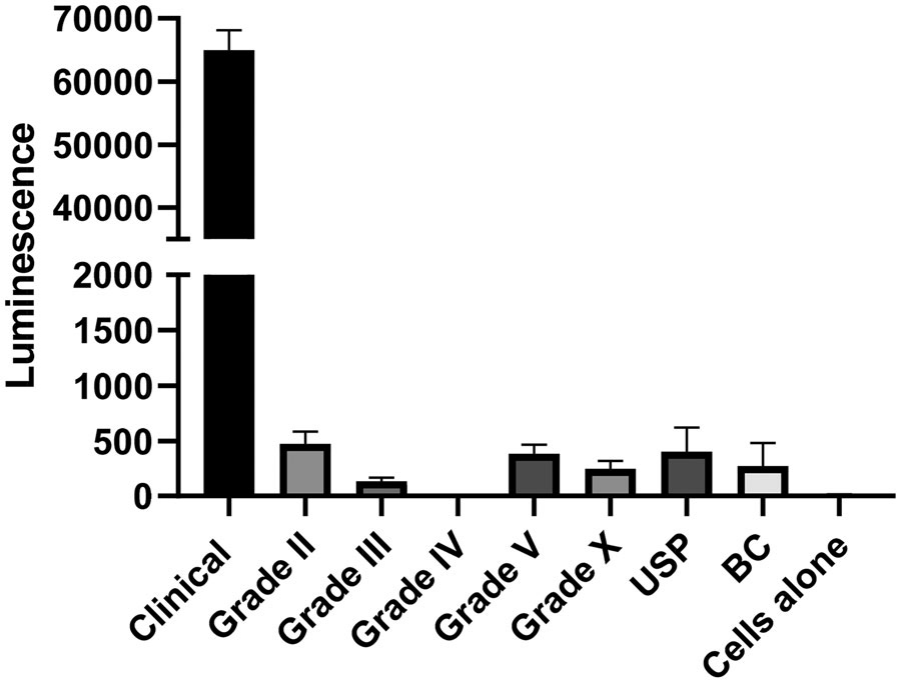
Transfection capacity in HEK cells. A total of 10^5^ cells per well were incubated for 20 hours with proticles (1 µg of RNA and 3 µg of protamine per well). Then, the relative amounts of luciferase produced were evaluated by the addition of luciferin and the measurement of light emission in a luminometer. Data represent triplicate mean values; error bars: SD. A t-test analysis shows that ‘clinical’ protamine is significantly better than all other protamines with *p* <.0001.

**Figure 5.**
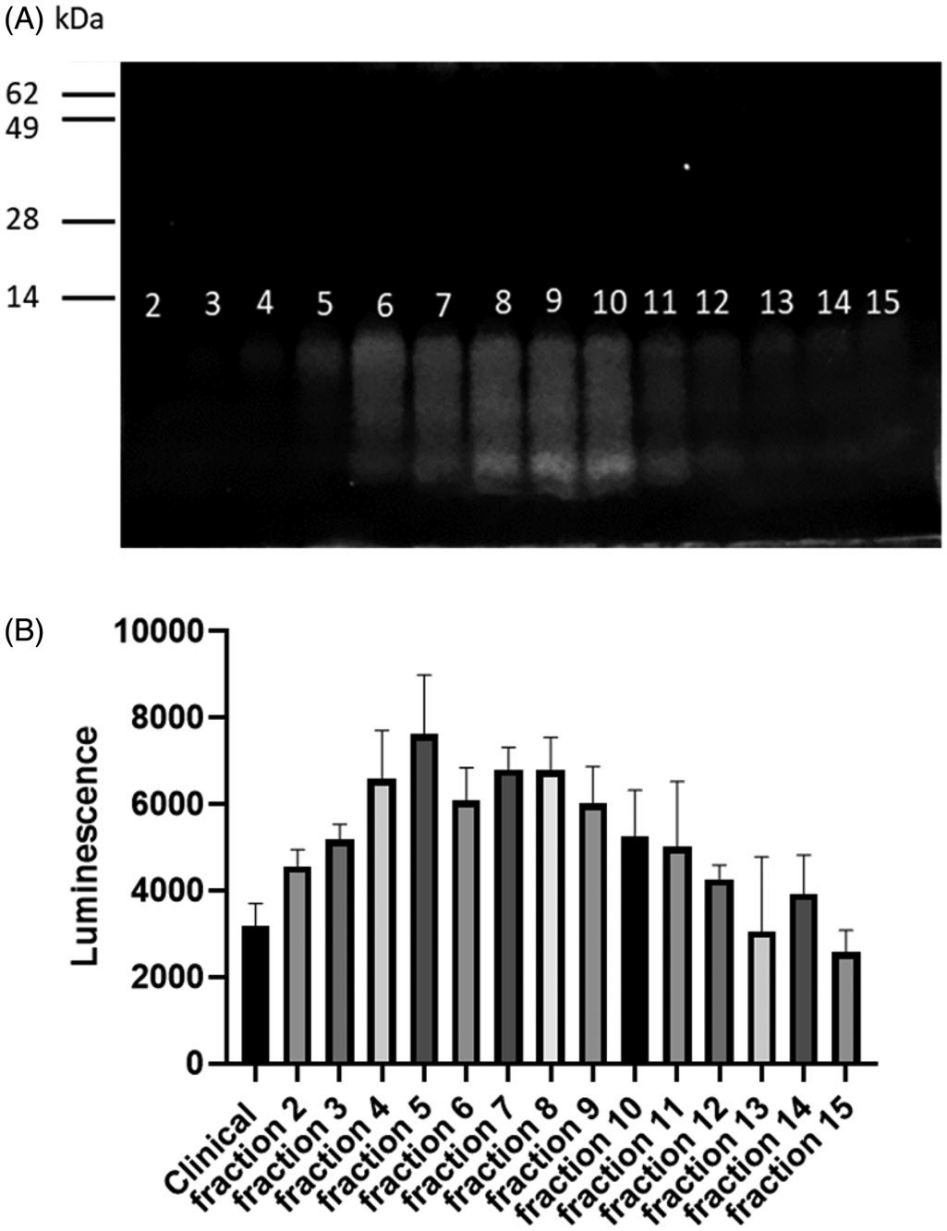
Fractionation of clinical protamine. (A) Visualization of the clinical protamine fractions on polyacrylamide gel. Among the 24 fractions collected, proteins were visible in fractions 2–15. (B) Transfection efficiency of nanoparticles formulated using the clinical protamine (‘clinical’ ) or each Sephadex fraction mixed with mRNA. HEK cells were incubated with nanoparticles (0.5 µg of mRNA and 1.5 µg of protamine) formed from the fractions (90 µg/ml) mixed with mRNA (30 µg/ml). After 18 h of incubation, the luminescence was measured. Data represent triplicate mean values; error bars: SD.

Finally, the cytosolic delivery capacity of all protamines was tested. To this end, we measured the translation of mRNA. Surprisingly, only the clinical chloride protamine 5000 produced a strong expression of the condensed mRNA at the 3:1 ratio (Figure 4). Increasing the protamine to RNA ratio could improve the transfection capacities of other protamines (Supplementary Figure 3). However, most protamines were relatively inefficient at all ratios (grade IV giving barely any detectable luciferase activity), while clinical chloride protamine 5000 remained the most efficacious one. This feature might be attributed to several functional characteristics of clinical chloride protamine 5000 such as the capacity to protect mRNA from degradation, to facilitate cellular uptake, to induce endosomal escape, or to enable mRNA dissociation from proticles when within the cytosol. Further mechanistic studies will be required to analyze those aspects in details. Meanwhile, to further characterize the type of protamines that allow transfection, we fractionated clinical chloride protamine 5000 on Sephadex P25 columns (Figure 5(A)) and tested the fractions in the mRNA transfection assay (Figure 5(B)). It appears that the large protamines contain the moieties that are critical to drive the expression of the condensed mRNA: fraction 5, which contains only the highest weight protamine, produced the best transfection result. This protamine results in expression levels more than two-fold higher than that observed with the full mixture of clinical chloride protamine peptides. Thus, new preparations of protamines that contain only the largest peptides may be optimal for the cytosolic delivery of RNA.

In summary, we show that various commercially available protamines differ in their appearance on SDS-PAGE and in capacity to effectively transfect mRNA into the cytosol. However, all tested protamines were equivalent in their ability to form nanoparticles when mixed with RNA and to deliver RNA to endosome resident TLRs. Protamines are frequently used to package nucleic acids (antisense, siRNA, immunostimulatory nucleic acids, plasmid DNA, or messenger RNA) (Hoerr et al., 2000; Scheel et al., 2005; Vighi et al., 2012; Weide et al., 2009; Feyerabend et al., 2009), and several such formulations are in clinical trials. Our work highlights the functional relevance of the protamine source in the development of pharmaceutical drugs and paves the way toward the development of standardized and optimized protocols for the delivery of nucleic acids.
